# Literary Note from the Century Company

**Published:** 1887-03

**Authors:** 


LITERARY NOTE FROM THE CENTURY COMPANY.—The sales of *The Century Magazine* have gone up over 30,000 copies in six weeks, since beginning the Life of Lincoln. A second edition of December was issued on the 15th. A veteran New York publisher predicts that the permanent edition of the magazine will go beyond 300,000 before the completion of the Lincoln history. The January installment, which is said by the editors to be of most surpassing interest, occupies thirty pages of the magazine, and treats of Mr. Lincoln's settlement in Springfield ; his practice of law in that city ; the Harrison campaign ; Lincoln's marriage ; his friendship with the Speeds of Kentucky ; the Shields duel, and the campaign of 1844. The illustrations are numerous, including portraits of Joshua Speed and wife, of Mrs. Lucy G. Speed, Milton Hay, President Harrison, General Shields, William H. Herndon (the law partner of Mr. Lincoln), and Mr. Lincoln himself, from the photograph presented by him to Mrs. Lucy G. Speed, in 1861. Pictures are given of the house where Lincoln was married, also of the house where he lived after his marriage, etc.

